# Evaluation of Serologic Changes of IgG and IgM Antibodies Associated with SARS-COV-2 in Cancer Patients: A Cohort Seroprevalence Study

**DOI:** 10.31557/APJCP.2021.22.6.1667

**Published:** 2021-06

**Authors:** Maliheh Arab, Somayyeh Noei Teymoordash, Maryam Talayeh, Behnaz Ghavami, Abdolreza Javadi, Behnaz Nouri

**Affiliations:** 1 *Department of Gynecology Oncology, Imam Hossein Medical Center, Shahid Beheshti University of Medical Sciences, Tehran, Iran. *; 2 *Obstetrician & Gynecologist, Fellowship of laparoscopy, Tehran, Iran. *; 3 *Department of Pathology and Laboratory Medicine, Imam Hossein Hospital, Shahid Beheshti University of Medical Sciences, Tehran, Iran. *; 4 *Department of Obstetrics and Gynecology, Shohada Tajrish Hospital, Shahid Beheshti University of Medical Sciences, Tehran, Iran. *

**Keywords:** COVID-19- SARS-CoV-2- cancer- oncology- asymptomatic

## Abstract

**Background:**

While the coronavirus disease 2019 (COVID-19) pandemic spreads, there is increasing evidence to suggest the elevated risk of SARS-CoV-2 infection and following morbidity and mortality in cancer patients. Serology testing using ELISA proposes major advantages as a diagnostic and preventive tool to control the present SARS-CoV-2 outbreak. This cohort study was to determine the SARS-CoV-2 seroconversion in asymptomatic cancer patients.

**Methods:**

Patients in all age groups and with any type of cancer who have been in remission or have stable disease and received their latest anticancer therapy over 2 months ago included in the study. All patients were evaluated for COVID-19 symptoms and only asymptomatic patients were enrolled for serologic screening for SARS-CoV-2. Serum samples evaluated serologically for SARS-CoV-2 antibodies by enzyme-linked immunosorbent assay.

**Results:**

A total of 168 asymptomatic cancer patients were included in the study. Of the 168 cases with a history of cancer who were asymptomatic for Covid-19, 29 cases (17.26%) had a positive serological test.

**Conclusion:**

In conclusion, in the present study asymptomatic cancer patients revealed 17% seropositivity, approximately equal to the general population of the same age, sex, geographic region, and epidemic status. Asymptomatic infections should further be investigated and considered as playing an important role in the COVID-19 transmission chain.

## Introduction

Prevalence of the novel coronavirus pneumonia began in Wuhan, China at the end of December 2019. This disease was officially termed as coronavirus disease 2019 (COVID-19) by the World Health Organization (WHO) on February 11, 2020 (Gao et al., 2021). Soon after COVID-19 outbreak in Wuhan, China, it became a pandemic which, so far, has infected more than 150 million people worldwide and death toll has been reported to reach 3,205,000 on May 03, 2021 in 188 countries and regions. As of May 3^th^, 2021, a total of 2,534,855 infected cases and 72,484 deaths had been recorded in Iran (medicine, 2020)

To date, no specific treatment has shown to be effective against COVID-19; thus, Performing the recommended prevention and control measures of infection along with supportive cares of complications have been the main goals (Al-Quteimat and Amer, 2020). However, with the global outbreak of coronavirus, it became evident that many COVID-19 cases are asymptomatic, though able to infect the others with virus. WHO defines an asymptomatic case as a laboratory-confirmed infected person, who doesn’t show classic clinical symptoms, and has no obvious abnormal findings, as well as on lung CT scan (WHO, 2020).

Performing testing based on symptomatology and exposure history alone is potentially a suboptimal approach in protecting the vulnerable cancer population, as transmission from asymptomatic carriers presents a serious risk. It is known that a substantial proportion of COVID-19 patients are asymptomatic and are missed via screening based on symptoms alone (Madariaga et al., 2020). The European Society for Medical Oncology propose microbiological SARS-CoV-2 screening before active anticancer therapy (Colombo et al., 2020). Early diagnosis of COVID-19 positive individuals and interrupt the routes of disease transmission are crucial steps to limit and control SARS-CoV-2 outbreak.

Although, PCR test is the gold standard for diagnosis, ≥14 days after infection, this test might be negative. As a result, the prevalence of the infection can be underestimated.

Serology testing using ELISA provides major advantages as a diagnostic and preventive tool to control the present pandemic and probably re-emergence of SARS-CoV-2 outbreak. Therefore, the evolution of manual ELISA test remains one of the most important consideration, since these can supplement the specific COVID-19 molecular test while solve some of its limitations (Zhou and Zhao, 2020).

Iran was among the highly infected countries in the global outbreak of SARS-CoV-2. Awareness of asymptomatic individuals, especially in subgroups such as cancer patients, can be of helpful in prevention of the disease spread in the community. We conducted a seroepidemiologic cohort study among asymptomatic cancer patients. The aim of our cohort study was to evaluate the SARS-CoV-2 seroconversion in asymptomatic cancer patients.

## Materials and Methods

Patients in all age groups and with any type of cancer who have been diagnosed during the last five years were evaluated for inclusion in this study. Also, to be eligible for this study, patients had to be in remission or have stable disease in which they have received their latest anticancer therapy over 2 months ago. Patients in active phase of the treatment were excluded from the study. All patients were evaluated for COVID-19 symptoms and only asymptomatic patients were enrolled for serologic screening for SARS-CoV-2. Demographic data were collected by filled questionnaires for each patient, obtaining relevant data. Serum samples evaluated serologically for SARS-CoV-2 antibodies by enzyme-linked immunosorbent assay with Pishtaz Teb SARS-CoV-2 ELISA kits approved by Iran Food and Drug Administration in the second peak of SARS-CoV-2 in Jun and July 2020. The study protocol was verified by the ethical committee of Shahid Beheshti University of Medical Sciences.

## Results

In the present study, 168 patients were enrolled. Ninety nine out of the 168 patients (56.4%) were more than 50 years old. The mean age and mean body mass index (BMI) of studied patients were 50.7 ± 11 years and 29±5/03, respectively. The most common cancers included breast cancer in 101 (60%), uterine cancer in 27 (16.1%), and cervical cancer in 17 (10.2%). Of the 168 patients with history of cancer who were asymptomatic for Covid-19, 29 cases (17.26%) had positive serological test, as follows: three patients (1.78%) were positive for both IgG and IgM, 24 patients (14.28%) were IgG positive and IgM negative, two patients (1.19%) were IgM positive and IgG negative.

**Table 1:** Comparison of Asymptomatic Positive Cases in Different Groups of Population

Author	Country	Studied Population	Mean Age	Type of Test	Rate of asymptomatic patients
Arab et al., (2020)	Tehran, Iran	Asymptomatic cancer patients in remission or stable phase (N=168)	50.73	Serological SARS‐CoV‐2 test	17.26%
Nekkanti et al., (2020)	Mumbai, India	Asymptomatic cancer patients (N=262)	48.5 year	RT-PCR	8%
Kimball et al., (2020)	Washington, USA	Older adults in skilled nursing facility (N=76)	80.70 year	RT-PCR	17.10%
He et al., (2020)	China and other countries	Meta-analysis included 41 studies (N=50155)	35.75 year (from 4 studies included in the meta-analysis)	RT-PCR or serological test	15.60%
Nishiura et al., (2020)	Japan	Japanese passengers of chartered flights evacuated from Wuhan (N=565)	─	RT-PCR	30.80%
Wu et al., (2020)	Wuhan, China	Hospitalized patients (N=380), permission of resume group (1021)	─	NAT and serological SARS‐CoV‐2 test	10.26% for hospitalized patients, 9.60% for resume group
Korth et al., (2020)	Essen, German	Healthcare workers of a tertiary hospital	High risk: 36.7 Low risk: 42.3	SARS-CoV-2-IgG antibodies	1.60%
Al-Shamsi et al., (2020)	Dubai, UAE	Asymptomatic patients with cancer (N=85)	55	RT-PCR	8%
Yin et al., (2020)	Wuhan, China	Patients with cancer in Hubei Cancer Hospital received anticancer treatment within the past 2 months (N=2818)	56.4	Serological SARS‐CoV‐2 test or RT-PCR	2.90%
Wang et al., (2020)	Wuhan, China	Non-critically ill individuals with positive SARS-CoV-2 RT-PCR tests (N=1012)	50	RT-PCR	3%

## Discussion

The certain diagnosis of COVID-19 is by the use of RT-PCR method to detect the SARS-CoV-2 virus from nasopharynx, saliva, or stool samples. However, RT-PCR has sensitivity of 70 to 80%, insufficient to be relied upon for conducting a strict strategy to control the spread of the infection. Meanwhile, the antibody testing is reported to be approximately 100% sensitive, although IgM or IgG antibodies turn positive 7 to 14 days after the symptomatic phase (Nagasawa et al., 2020). It is noteworthy that the RNA-based molecular tests are expensive and technically sophisticated investigative tools, and require high level lab facilities and strict protection of personnel. On the other hand, serology tests are easily performed in the most laboratories, thus are more widely applied than the molecular tests (Xiao et al., 2020).

Distribution of positive serologic or PCR tests in different asymptomatic subgroups in some studies are presented in Table 1 with a range of 1.6-30%. 

In the present study, 17% of asymptomatic cancer patients during remission were seropositive. Geographical region, time of study related to epidemic peak, sex, and age of individuals play a role in the seroconversion of asymptomatic individuals. In the study by Poustchi et al., (2021), females were more likely to be seropositive. With increasing age, this probability increases so that in the age group of 30-39 years, seroconversion rate was 11.8% and in the age group of 50-59 years, it was 14.7%. On the other hand, the seroconversion rate is different in geographical areas. This rate was 16.3% in the general population and 22.3% in high-risk occupations at the time of the first COVID-19 peak in Tehran (Poustchi et al., 2021)

The difference in seropositivity prevalence in different studies might be due to above mentioned issues (Table 1). Seropositivity in asymptomatic cancer patients at the time of the present study in the second peak of the COVID-19 pandemic in June and July in Tehran was 17%. Compared to the study conducted by Poustchi (2021), the rate of seroconversion during the first peak of COVID-19 pandemic in the general population and high-risk occupations in Tehran was 16.3% and 22.3%, respectively.

Cancer patients don’t appear to have a higher risk of seroconversion in comparison to the general population; Considering that in the present study the mean age of the studied population was 50.7 years. It seems that these cancer patients who were in the remission period, probably took more care of themselves due to their previous history of cancer, and also, they were mostly housewives, that is; they did not have any occupational contact. On the other hand, serological changes are indicative of infection. The risk of infection and seroconversion in asymptomatic cancer patients was not higher than other subgroups in different studies (Table 1). However, in the case of symptomatic infection in cancer patients, the possibility of mortality might be higher. In this subset of patients, there is a 3.5-fold increase in the risk of mechanical ventilation requirement, ICU admission and death compared to general population, due to immunosuppression resulting from the malignancy itself and anticancer treatments, like chemotherapy or surgery (Al-Quteimat and Amer, 2020; Kuderer et al., 2020; Lara et al., 2020; Liang et al., 2020). In the articles, surgery was shown to be related with worse clinical outcomes and increased postoperative mortality in COVID- positive patients (Nekkanti et al., 2020)

In conclusion, in the present study asymptomatic cancer patients revealed 17% seropositivity, approximately equal to the general population of the same age, sex, geographic region and epidemic status. Asymptomatic infections should further be investigated and considered as playing an important role in the COVID-19 transmission chain.

## Author Contribution Statement

Study conception and design: MA; data collection: SNT. MT. AJ; analysis and interpretation of results: MA. SNT. BG. draft manuscript preparation: MA. SNT. AJ. BN; All authors reviewed the results and approved the final version of the manuscript.
